# Impact of multiple policy interventions on the screening and diagnosis of drug-resistant tuberculosis patients: a cascade analysis on six prefectures in China

**DOI:** 10.1186/s40249-021-00793-9

**Published:** 2021-01-19

**Authors:** Xiao-Yan Ding, Wen-Hui Mao, Wei Lu, Hao Yu, Qiao Liu, Peng Lu, Hui Jiang, Xing Zhang, Feng Lu, Jie Xu, Chong-Qiao Zhong, Jin-Liu Hu, Wei-Xi Jiang, Lei Guo, Sheng-Lan Tang, Li-Mei Zhu

**Affiliations:** 1 Jiangsu Provincial Center for Disease Control and Prevention, 172 Jiangsu Road, Nanjing, 210009 Jiangsu China; 2 Duke Global Health Institute, Duke University, 310 Trent Dr, Durham, NC 27710 USA; 3 Zhenjiang Center for Disease Control and Prevention, No. 9 South Huangshan Road, Zhenjiang, 212000 Jiangsu China; 4 Changzhou Center for Disease Control and Prevention, No. 28 Jianshen Road, Changzhou, 213000 Jiangsu China; 5 Nantong Center for Disease Control and Prevention, No. 189 South Gongnong Road, Nantong, 226007 Jiangsu China; 6 Yangzhou Center for Disease Control and Prevention, No. 36 East Yanfu Road, Yangzhou, 225001 Jiangsu China; 7 Lianyungang Center for Disease Control and Prevention, No. 161 Middle Hailian Road, Lianyungang, Jiangsu China; 8 Huai’an Center for Disease Control and Prevention, No. 6 Meigao Road, Huaian, 223001 Jiangsu China; 9 Duke Kunshan University, Kunshan, Jiangsu China

**Keywords:** Drug-resistant tuberculosis, Policy impact, Screen, Diagnosis, China

## Abstract

**Background:**

The detection of drug-resistant tuberculosis (DR-TB) is a major health concern in China. We aim to summarize interventions related to the screening and detection of DR-TB in Jiangsu Province, analyse their impact, and highlight policy implications for improving the prevention and control of DR-TB.

**Methods:**

We selected six prefectures from south, central and north Jiangsu Province. We reviewed policy documents between 2008 and 2019, and extracted routine TB patient registration data from the TB Information Management System (TBIMS) between 2013 and 2019. We used the *High-quality Health System Framework* to structure the analysis. We performed statistical analysis and logistic regression to assess the impact of different policy interventions on DR-TB detection.

**Results:**

Three prefectures in Jiangsu introduced DR-TB related interventions between 2008 and 2010 in partnership with the Global Fund to Fight AIDS, Tuberculosis and Malaria (the Global Fund) and the Bill & Melinda Gates Foundation (Gates Foundation). By 2017, all prefectures in Jiangsu had implemented provincial level DR-TB policies, such as use of rapid molecular tests (RMT), and expanded drug susceptibility testing (DST) for populations at risk of DR-TB. The percentage of pulmonary TB cases confirmed by bacteriology increased from 30.0% in 2013 to over 50.0% in all prefectures by 2019, indicating that the implementation of new diagnostics has provided more sensitive testing results than the traditional smear microscopy. At the same time, the proportion of bacteriologically confirmed cases tested for drug resistance has increased substantially, indicating that the intervention of expanding the coverage of DST has reached more of the population at risk of DR-TB. Prefectures that implemented interventions with support from the Global Fund and the Gates Foundation had better detection performance of DR-TB patiens compared to those did not receive external support. However, the disparities in DR-TB detection across prefectures significantly narrowed after the implementation of provincial DR-TB polices.

**Conclusions:**

The introduction of new diagnostics, including RMT, have improved the detection of DR-TB. Prefectures that received support from the Global Fund and the Gates Foundation had better detection of DR-TB. Additionally, the implementation of provincial DR-TB polices led to improvements in the detection of DR-TB across all prefectures. 
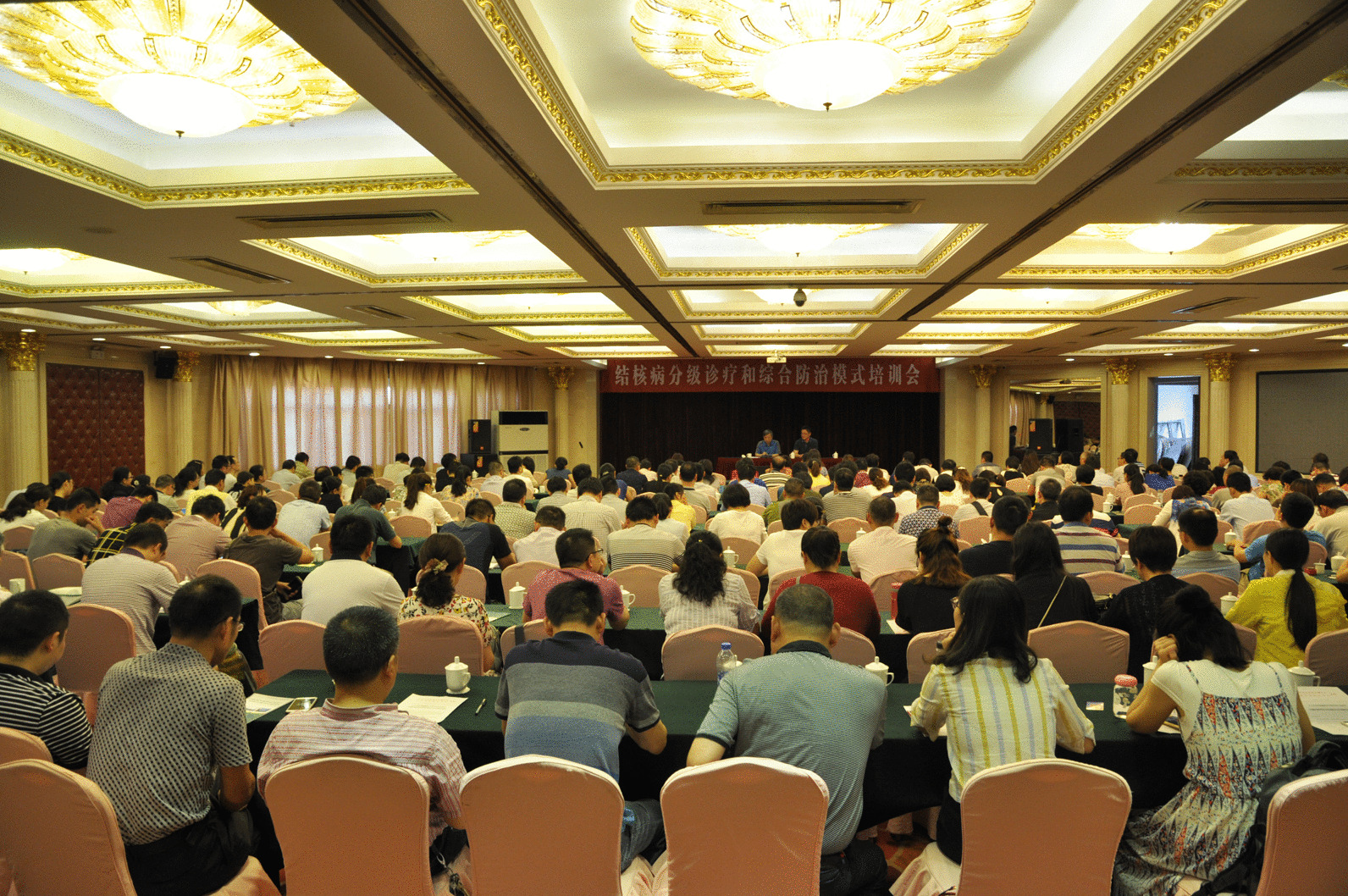

## Background

Tuberculosis (TB) is a communicable disease that remains a key global health concern. In 2017, TB led to the highest number of deaths and the second highest disability-adjusted life years (DALYs) among all communicable diseases globally [1]. The End TB Strategy led by the World Health Organization endeavours to reduce TB deaths by 95% and eliminate catastrophic health expenditures caused by TB by 2035 [2].

China has made great achievements in TB care and control. China’s incidence of TB has been declining in recent decades, from 170/100,000 in 1990 to 61/100,000 in 2018 [3]. However, drug-resistant TB (DR-TB) continues to remain a public health threat in China. A 2007 national survey reported that 5.7% of new TB patients and 25.6% of previously treated TB patients were infected with multidrug-resistant TB (MDR-TB) [4]. Compared to regular TB, MDR/rifampicin-resistant(RR)-TB is more resource intensive and has a longer treatment course. Globally, the treatment success rate for RR-TB is not even 60% [5, 6]. In 2018, less than one fourth of MDR/RR-TB patients around the world were estimated to be detected, and of those that were detected, only 60% had initiated treatment [6].

Multiple strategies have been implemented in China to increase the detection of and treatment rates for DR-TB patients. In 2006, China started a partnership with the Global Fund to Fight AIDS, Tuberculosis and Malaria (the Global Fund) in two provinces. The partnership aimed to improve DR-TB diagnosis, treatment, and management [7]. Through another partnership in 2009 with the Bill & Melinda Gates Foundation (BMGF), rapid molecular tests (RMT) for DR-TB diagnosis and other treatment and financing interventions were piloted in four prefectures across four provinces in China [8, 9]. Given its rapid economic development, the Global Fund ended its partnership with China in 2014 and as a response to the end in external support, the Chinese government put more funding and policy support into DR-TB control and care. For example, both the twelfth and thirteenth national “Five-year” plans for TB control set goals to improve DR-TB control and care. In particular, these plans called for the establishment of a new financing mechanism for DR-TB control, regulation of the use of second line TB drugs, establishment of prefecture-level treatment and management sites, improved health services for DR-TB patients, and enhanced capacity building for DR-TB case-finding [7, 10].

In China, TB strategies have historically been implemented at the provincial level. However, there is great variation among provinces, particularly between those that received external funding from donors and those that did not. There was certain evidence gap in reporting the various TB strategies across different regions. Additionally, multiple TB policies were implemented at the same time and jointly influence the detection and care for DR-TB. Unfortunately, most of the existing evidence assesses a single policy intervention, while none have yet evaluated the impact of multiple interventions. Wang et al. summarized the implementation of projects funded by the Global Fund across China, but there was no assessment on the impact of different interventions [7]. Li et al. evaluated the effect of a comprehensive DR-TB project with focus on access to diagnosis, quality treatment, and affordable treatment for MDR-TB [8]. Huang et al. evaluated the benefit of expanding the diagnostic algorithm for diagnosis and treating bacteriologically confirmed pulmonary TB and RR-TB [11]. Considering the significant variance in DR-TB policies across the country and the various external factors beyond the health sector that may also influence DR-TB control and care, it is essential to understand the complexity of DR-TB interventions to further improve the DR-TB policies at both the national and provincial level.

The purpose of this study is to summarize the interventions and policy changes in the screening and detection of DR-TB patients in Jiangsu Province, analyse their impact, and make recommendations for the prevention and control of DR-TB in other contexts. Jiangsu has a long history of DR-TB control through both partnership with international organizations and domestic efforts, and it performs among the highest across all provinces in China for the detection of DR-TB. By examining DR-TB policies and their impact using real-world evidence from Jiangsu, useful insights on DR-TB control could be extended to other regions of China.

## Methods

### Settings

We selected Jiangsu Province as our study site. Jiangsu lies on the east coast of China. It is the fourth wealthiest province in China, with a GDP per capita of USD 17 450 in 2018. There are thirteen prefectures in Jiangsu Province with populations ranging from 3.2 million to 10.7 million. There is regional variance in economic development across the province, with prefectures in southern Jiangsu being the most developed followed by those located in central and northern Jiangsu. In general, more developed regions of Jiangsu have better TB detection and care performance.

We selected Jiangsu Province for several reasons. First, Jiangsu hosted one of the first project sites for international collaboration on DR-TB control and care, including collaborations with the Global Fund and BMGF. Second, the provincial and prefecture-level governments of Jiangsu were very supportive of the prevention and treatment for DR-TB. Jiangsu’s provincial TB control policies generally had higher requirements than those outlined in the national “Five-year” plans. Finally, Jiangsu Province has had a digitized Hospital Information System (HIS) and TB Information Management System (TBIMS) since 2005 [12]. The accessibility and quality of historical data made our analysis feasible.

We selected two prefectures from each region of Jiangsu Province: Zhenjiang and Changzhou (South), Nantong and Yangzhou (Central), Huai’an and Lianyungang (North). These prefectures were selected for several reasons, including data availability and quality, diversity of development status, and variation among TB policies. Socioeconomic information of selected prefectures can be found in Additional file 1: Socioeconomics in study sites.

### Conceptual framework

We used the *High-quality Health System Framework* to structure the analysis [13]. We first looked at the *foundations* that have been funded and used in DR-TB prevention and treatment in Jiangsu (e.g., policy, workforce, diagnostic devices). Then, we described the *processes* of care used for DR-TB testing, such as turnaround time for testing results. Finally, we presented the percentage of pulmonary TB cases confirmed by bacteriology and the percentage of bacteriologically confirmed TB patients tested for drug susceptibility as the *impact* indicators of the DR-TB interventions.

### Data collection

We collected government policy documents related to prevention, screening, diagnosis, and management of DR-TB. We also collected protocols of domestically and internationally funded DR-TB related projects from 2008 to 2019 at the provincial, prefecture, and county levels to understand the interventions by project funders as well as the policies issued by local government. We collected data on notified TB cases and presumptive DR-TB patients from the TB Information Management System (TBIMS) [12]. Data were restructured into three case-based databases: one for all active TB cases, one for patients with presumptive DR-TB, and one for confirmed DR-TB cases.

We extracted notified TB case records and presumptive DR-TB case records from TBIMS from the six selected sites. Records with a diagnosis of “pulmonary tuberculosis” between January 1, 2013 and December 31, 2019 were included for analysis. All other types of TB, such as bone tuberculosis, extrapulmonary tuberculosis were excluded. Therefore, all results presented in this article refer to pulmonary TB only.

We collected the following indicators for notified TB patients (i.e., those with pulmonary TB) and presumptive DR-TB patients: registration number, age, sex, residency (e.g., within the county, within the prefecture), and TB related information including bacteriological results, previous TB treatment, whether diagnosed at county or prefecture agency, type of diagnosis agency, and date of entry into TBIMS. Similarly, we collected the following indicators for presumptive DR-TB patients: diagnosis region, registration number, age, sex, source of presumptive DR-TB (e.g., linked with TB database, directly registered and linked with confirmed DR-TB database), the registration date of basic management unit (BMU) [14, 15], smear results, culture results, strain identification results, types of drug susceptibility testing (DST) (phenotypic or genotypic), results of DST, confirmed date of DST, and date of entry. No personally identifiable information was extracted from the database.

### Data analysis

Descriptive analysis on key indicators was disaggregated by year and by prefecture. The definitions of key indicators of interest are listed below.

1. Bacteriologically confirmed TB case: A patient from whom a biological specimen is positive by smear microscopy, culture, or rapid diagnostic test.
2. Percentage of pulmonary TB cases confirmed by bacteriology: The number of bacteriologically confirmed TB cases divided by the number of cases diagnosed with TB cases.
3. Percentage of bacteriologically confirmed TB patients tested for drug susceptibility: The number of bacteriologically confirmed TB cases tested for drug resistance (including those tested only for rifampicin resistance) divided by the number of bacteriologically confirmed TB cases.
4. Suspected high risk (M)DR-TB patients [7]: TB patients with at least one of the following conditions: (a) failure to respond to initial TB treatment as new TB patient (for new smear positive patients, smear or culture positive after five months of treatment; for new smear negative patients, smear or culture positive for any time during treatment), (b) failure to respond to the most recent retreatment as relapse TB case, (c) chronic cases of TB, (d) close contact with a known MDR-TB patient, (e) smear-positive at the end of 2 to 3 months of initial treatment.
5. Testing time for the diagnosis of DR-TB: days from the registration date in BMU to the confirmed date of DST.

We used logistic regression models on notified TB records to further assess the impact of different policy interventions. In model 1, the dependent variable was the bacteriological results. The key explanatory variable was use of the new diagnostic devices. The control variables were: year of registry, prefecture, sex, age group (e.g., under 45, 45 to 60, and over 60), treatment history (e.g., new patient, previously treated patient), cavitary disease (e.g., no, yes), administrative level of diagnosis agency (e.g., prefecture, county), type of diagnosis agency [e.g., general hospital, specialized hospital, clinic or TB dispensary of Center for Disease Control and Prevention (CDC)], and current residency (e.g., within county, within prefecture, within Jiangsu, outside Jiangsu). For model 2, the dependent variable was confirmed TB patients tested for drug susceptibility. We used a group of dummy variables as a key explanatory variable to indicate the interventions on DST coverage, while the control variables were the same as model 1.

We performed analysis in IBM SPSS Statistics 22.0 (Armonk, NY, USA).

## Results

### Policies and major interventions

**Fig. 1:**
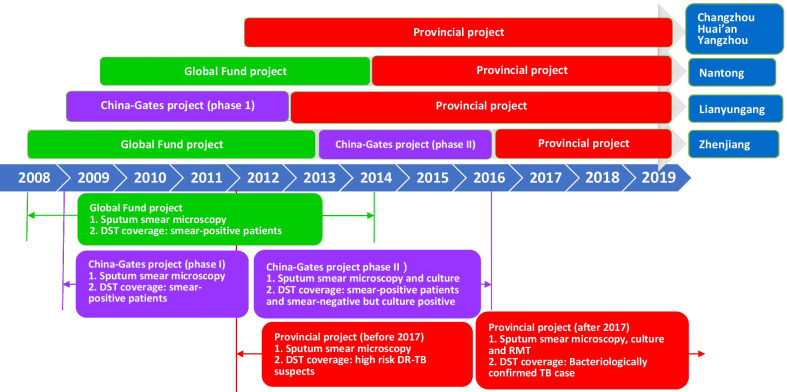
Timeline of major policies and interventions on the screening and diagnosis of DR-TB in Jiangsu, 2008–2019. Notes: DST: drug susceptibility testing; DR-TB: drug-resistant tuberculosis; RMT: rapid molecular tests; Global Fund project: Global Fund to Fight AIDS, Tuberculosis and Malaria

Based on our review of relevant policy documents and intervention protocols, we mapped out different TB interventions that were implemented in each prefecture between 2013 and 2019. We summarize all DR-TB related interventions between 2008 and 2019 (Fig. 1) including the timeline, scope, and implementation sites of major interventions.

Zhenjiang was the first prefecture to implement DR-TB interventions on treatment and patient management (Global Fund, 2008–2013), followed by Lianyungang (Gates Foundation, 2009–2012) and Nantong (Global Fund, 2009–2014). These three prefectures started to follow provincial level DR-TB interventions after the end of their respective internationally funded projects. Conversely, Changzhou, Huai’an, and Yangzhou implemented DR-TB interventions relatively late, beginning only in 2012. These three prefectures did not host any internationally funded TB-related projects, and therefore only implemented provincial level DR-TB interventions.

The main interventions supported by the Global Fund project from 2008 to 2014 in Jiangsu (Fig. 1) included: (i) DST with smear-positive patients; (ii) covering the cost of hospital admission for MDR-TB treatment; (iii) providing MDR-TB patients with a transport subsidy; and (iv) ensuring a consistent supply of second-line drugs. The first phase of the China-Gates project focused on improving MDR/RR-TB diagnosis, treatment, financing, and DST with smear positive patients. The second phase of the China-Gates project focused on setting up a comprehensive TB control model for effective diagnosis, treatment, and management of TB patients using the molecular diagnosis technology and expanding DST coverage [16]. Provincial-funded projects focused on diagnosis, treatment, management, financing of MDR-TB patients, and DST with suspected high risk (M)DR-TB patients in its initial phase. To accelerate the pace of TB control, RMT was introduced to all TB labs by 2017 and DST was expanded to all bacteriologically confirmed TB cases.

### Workforce and funding for DR-TB

We reviewed and compared the changes in TB related staff, funding for DR-TB, and laboratory equipment between 2013 and 2018 as the input of DR-TB detection.

Although the number of designated TB hospitals remained the same for most prefectures between 2013 and 2018 (with the exception of Zhenjiang), the number of RMT devices increased substantially, indicating more TB designated hospitals were outfitted with RMT devices (Additional file 1: Workforce and funding for TB). In 2013, there were only three TB laboratories equipped with RMT devices. By 2018, all TB laboratories were equipped with RMT devices, funded either by international programs or the provincial government.

Between 2013 and 2018, the total number of full-time staff at local CDCs dropped slightly. Full-time staff of local CDCs declined in five prefectures, with the greatest declines found in Zhenjiang and Nantong. Staff of the designated TB hospitals increased during this same time period, mainly because the TB care model shifted from CDC to designated TB hospitals. Between 2013 and 2018, full-time staff of designated TB hospitals increased in most prefectures, among which Yangzhou saw the most growth (from 32 to 71).

Funds allocated specifically for DR-TB control and prevention from the provincial government increased substantially in five prefectures. The disparity of special funds dedicated to DR-TB narrowed in 2018: Lianyungang was the only prefecture that saw a decline in special TB funds and Nantong had the most rapid growth, followed by Zhenjiang and Huai’an (Additional file 1: Workforce and funding for TB).

### Testing approach and process

Testing approaches and processes are regarded as intermediate changes to DR-TB detection practice. As mentioned in the previous sections, more devices have been put into use in Jiangsu while the target population screening for DST has been expanded. We compared the testing approach and process for DR-TB between 2013 and 2019 to observe changes.

Conventional diagnosis methods (including sputum smear microscopy, culture and phenotypic DST) were mainly used before 2017 (Additional file 1: Process chart for TB testing in Jiangsu: 3a. Process chart with proportion method before 2017). Sputum smear microscopy, culture testing, and risk assessment for drug-resistance were previously performed at the county-level. Clinical isolates or sputum samples with positive results then had to be transferred from the county-level to the prefecture-level laboratory for DST. In contrast, the rapid molecular DST was available at the prefecture-level for those prefectures implementing international projects (e.g., Zhenjiang, Lianyungang, and Nantong.)

After the implementation of comprehensive provincial DR-TB control policies in 2017, RMT on sputum samples is performed at the county level in all prefectures across Jiangsu. Specifically, for counties equipped with Xpert MTB/RIF (Xpert, Cepheid, USA) in Zhenjiang and Nantong; RMT was performed on all TB cases while those equipped with loop-mediated isothermal amplification (LAMP, Deaou Biotechnology, China) in Changzhou, Lianyungang, Huai’an, and Yangzhou; RMT was performed on TB cases with smear-negative results. Since 2017, sputum samples and clinical isolates were transferred to the prefecture-level laboratory for DST. Charts showing the change in approach can be found in Additional file 1: Process chart for TB testing in Jiangsu: 3b–3d.

### Testing time

**Fig. 2:**
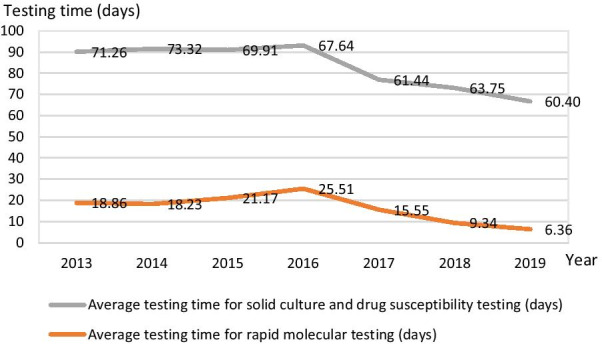
Average testing time for the diagnosis of DR-TB (days) in six prefectures of Jiangsu, 2013–2019

Testing time is jointly influenced by the type of test used and the testing process. Rapid turnaround time for testing can bring timely treatment and reduce potential risk of spreading infection. RMT has a significantly shorter time compared with solid culture and DST. From 2013 to 2019, the average time for RMT shortened by two thirds (from 18.86 to 6.36 days) while the average time for solid culture and DST dropped slightly, but still remained over 60 days. All prefectures saw similar trends in reduction of testing time for DST and RMT between 2013 and 2019 (Fig. 2).

### Percentage of pulmonary TB cases confirmed by bacteriology

The percentage of pulmonary TB cases confirmed by bacteriology increased from around 30.0% in 2013 to 55.0% in 2019. The application of various detection methods, such as culture and rapid TB testing resulted in the increase of the percentage of pulmonary TB cases confirmed by bacteriology (Table 1). Prefectures that implemented international projects generally had better performance than those that did not host international projects between 2013 and 2016. For instance, Zhenjiang, one site for a Gates Foundation project, had the highest percentage of pulmonary TB cases confirmed by bacteriology among the six selected prefectures. Most prefectures experienced significant improvement between 2017 and 2018 given the implementation of provincial-level policies in all prefectures (including the application of culture and RMT for suspected TB patients). The percentage of pulmonary TB cases confirmed by bacteriology in our six focus prefectures all reached 50.0% by 2019 while the disparities among prefectures gradually narrowed over the seven year time period reviewed (Table 1).

**Table 1:** Percentage of pulmonary TB cases confirmed by bacteriology and percentage of bacteriologically confirmed TB patients tested for drug susceptibility, disaggregated by prefectures, and year

Prefecture	Percentage of pulmonary TB cases confirmed by bacteriology (%)							Percentage of bacteriologically confirmed TB patients tested for drug susceptibility (%)						
	2013	2014	2015	2016	2017	2018	2019	2013	2014	2015	2016	2017	2018	2019
Zhenjiang	31.2	39.2	39.0	40.2	45.5	55.2	56.0	89.0	89.8	86.1	84.8	100.0	95.9	97.0
Changzhou	32.4	29.8	31.4	32.2	32.6	51.1	58.2	91.4	88.6	83.0	84.9	100.0	91.5	96.9
Huai’an	25.1	26.3	30.6	31.9	36.4	43.7	51.9	8.2	8.7	9.5	25.5	53.4	98.3	99.6
Lianyungang	29.4	25.5	28.4	32.4	41.5	50.5	57.3	88.8	92.6	90.5	87.9	87.0	87.7	93.5
Nantong	27.0	26.7	25.4	38.2	30.1	51.3	55.3	94.5	76.1	73.7	96.3	57.4	95.7	97.6
Yangzhou	32.8	30.9	32.2	34.6	37.7	52.6	54.4	25.4	14.6	22.7	30.8	62.9	94.8	98.0

### Percentage of bacteriologically confirmed TB patients tested for drug susceptibility

In 2013, the percentage of bacteriologically confirmed TB patients tested for drug susceptibility varied across the prefectures, from below 10.0% to over 90.0%. For prefectures that either performed DST on wider suspected DR-TB patients or performed DST before 2017, the proportion of bacteriologically confirmed TB patients tested for drug susceptibility was higher. The proportion of bacteriologically confirmed TB patients tested for drug susceptibility has risen sharply. This rise is particularly due to prefectures that had low testing rates prior to the implementation of provincial-level policies in 2017. In 2019, the percentage of bacteriologically confirmed TB patients tested for drug susceptibility was around 95% for all prefectures (Table 1).

### Influential factors for DR-TB screening and detection

Factors associated with pulmonary TB cases confirmed by bacteriology were analysed (Table 2). After controlling for demographic factors (e.g. TB history, residency and diagnosis agency), we found that the application of RMT (Xpert) is 1.11 times (*P* < 0.01) more likely to confirm TB cases than the smear test. The overall likelihood of pulmonary TB cases confirmed by bacteriology improved between 2013 and 2019. Females, the elderly (i.e., over 60), previously treated TB patients (compared with new patients), those with a cavity in a lung, and those residing outside the prefecture (compared with within county) are more likely to be bacteriologically confirmed. Patients are more likely to be bacteriologically confirmed by a county level diagnosis agency (compared with prefecture-level) and at general hospitals (compared with specialized hospitals, CDC, or TB dispensary).

**Table 2:** Influential factors for pulmonary TB cases confirmed by bacteriology and bacteriologically confirmed cases tested for drug resistance

Variables	Model 1 TB cases confirmed by bacteriology				Model 2 Bacteriologically confirmed cases tested for drug resistance			
	β	S.E	*P* value	*OR*	β	S.E	*P* value	*OR*
Smear test as base								
Rapid molecular testing	0.107	0.027	< 0.001	1.113	/	/	/	/
DST coverage: suspected high-risk DR-TB patients as base								
Smear positive TB patients	/	/	/	/	0.931	0.042	< 0.001	2.537
Smear positive or culture positive TB patients	/	/	/	/	0.834	0.042	< 0.001	2.304
Biologically confirmed TB patients	/	/	/	/	1.345	0.043	< 0.001	3.839
Year	0.174	0.005	< 0.001	1.190	0.196	0.009	< 0.001	1.217
Prefecture: Zhenjiang as base								
Changzhou	− 0.139	0.032	< 0.001	0.870	− 0.030	0.035	0.395	0.971
Huai’an	− 0.273	0.035	< 0.001	0.761	− 0.491	0.043	< 0.001	0.612
Lianyungang	0.014	0.042	0.734	1.014	0.016	0.051	0.759	1.016
Nantong	− 0.312	0.027	0.000	0.732	− 0.410	0.034	< 0.001	0.664
Yangzhou	− 0.109	0.035	0.002	0.897	− 0.212	0.044	< 0.001	0.809
Gender: male as base								
Female	0.144	0.017	< 0.001	1.155	0.248	0.021	< 0.001	1.281
Age group: over 60 as base (years)								
< 45	− 0.407	0.019	< 0.001	0.666	− 0.335	0.022	< 0.001	0.715
45–60	− 0.292	0.019	< 0.001	0.747	− 0.250	0.022	< 0.001	0.779
Treatment: new as base								
Retreatment	0.721	0.022	< 0.001	2.057	0.888	0.024	< 0.001	2.430
Cavitary disease: no as base								
Yes	0.078	0.037	0.034	1.081	0.052	0.041	0.206	1.053
Administrative level of diagnosis agency: county level as base								
Prefecture level	− 0.087	0.029	0.003	0.916	− 0.304	0.033	0.000	0.738
Type of current diagnosis agency: general hospitals as base								
Specialized hospitals	− 0.092	0.024	< 0.001	0.912	− 0.087	0.027	0.001	0.916
CDC or TB dispensary	− 0.221	0.035	< 0.001	0.802	− 0.247	0.043	< 0.001	0.781
Current residency: within county as base								
Within prefecture	0.001	0.025	0.965	1.001	− 0.150	0.029	< 0.001	0.861
Within province	0.344	0.060	< 0.001	1.410	− 0.456	0.082	< 0.001	0.634
Outside province	0.346	0.082	< 0.001	1.414	− 0.214	0.102	0.036	0.808
Constant	− 1.074	0.057	< 0.001	0.342	− 397.045	17.935	< 0.001	0.000
− 2 Log likelihood	98,704.781				77,168.226			
Cox & Snell R Square	0.066				0.135			
Nagelkerke R Square	0.090				0.200			

“/” means indicator not included in the model; *OR*: odd ratio

We analysed factors associated with bacteriologically confirmed cases tested for drug resistance (Table 2). After controlling for demographic factors (e.g., TB history, residency and diagnosis agency), our findings show that the following types of patients were significantly more likely to be tested for drug resistance: patients with DST coverage of biologically confirmed TB cases (3.84 times), smear-positive TB patients (2.54 times), and smear-positive TB patients and smear-negative but culture-positive TB patients (2.30 times). Females, the elderly (over 60), previously patients treated (compared with new patients), and those residing within the county (compared with outside the county), were more likely to be tested for drug resistance. Bacteriologically confirmed cases were more likely to be tested by a county level diagnosis agency (compared with prefecture-level) and at general hospitals (compared with specialized hospitals, CDC, or TB dispensary) for drug resistance.

## Discussion

Our study found that Jiangsu Province has implemented a series of interventions to improve DR-TB control and prevention, such as workforce increases, the use of RMT devices, and allocation of special funds focused on DR-TB. With the application of RMT, the diagnostic process of DR-TB has become more convenient to patients due to reduced testing time. The percentage of pulmonary TB cases confirmed by bacteriology has increased from around 30.0% in 2013 to over 50.0% in 2019, indicating that the implementation of new diagnostics, including RMT, have provided testing results more than the traditional smear microscopy. At the same time, the proportion of bacteriologically confirmed cases tested for drug resistance has increased substantially, indicating that the intervention of expanding the coverage of DST has reached more populations at risk of DR-TB.

Although there is no similar research analysing the impact of multiple DR-TB interventions on diagnosis, our findings are consistent with results from other evaluation studies on single DR-TB interventions, such as the implementation of RMT. For example, in Armenia the introduction of RMT increased the detection rate of RR-TB [17]; Oxlade et al. found that the implementation of rapid DST at the moment of diagnosis was the most cost-effective strategy [18]; and a study in South Africa found that the implementation of molecular DST reduced the laboratory turnaround time from 55 to 27 days [19]. These studies also found the same results as we did: the application of rapid DST has improved the diagnosis of DR-TB and that improved diagnosis is critical for achieving desired treatment results [20].

We observed that the three prefectures that participated in a Global Fund project and/or China-Gates Foundation project (Zhenjiang, Lianyungang, and Nantong) generally performed better than other prefectures, even after the partnerships concluded. This finding aligns with other studies. For example, in Nicaragua Plamondon et al. also found that the presence of a Global Fund project improved TB control, built human resource capacity, and strengthened community involvement in TB control [21]. Our findings signal that support from the Global Fund or the Gates Foundation had a strong emphasis on capacity building. Whether their support was for diagnostic devices or other best practices, such as DST to smear positive TB patients, the capacity building from international projects has had a long-lasting role in promoting the diagnosis for DR-TB [8].

Another significant finding is that the disparity in diagnosis for DR-TB among the six prefectures disappeared after 2017. This improvement can be attributed to the strong political commitment of the Jiangsu government in TB control and care. The provincial DR-TB intervention policies that were implemented were accompanied by sufficient resources such as staff, funding, and diagnostic devices, which ensured the sound implementation of interventions. In addition to the infrastructure acquired from international projects, Jiangsu Province made a significant investment to strengthen the infrastructure and capacity of laboratories of the prefectures that did not have international partnerships. For instance, both conventional equipment and RMT devices were introduced to all prefectures in Jiangsu (GeneChip to prefectures and LAMP to counties). In comparison, throughout the country only 44.2% of labs at the prefecture level and 27.5% at the county level have the same capacity as Jiangsu [22].

The diagnostic algorithm substantially improved after the application of RMT. The new process is more effective for the diagnosis of TB and DR-TB and reduces testing time, enabling patients to receive more timely treatment. While the overall testing time was reduced, we noticed that in 2016, testing time for RMT (Hain or GeneChip) was slightly longer than previous year after its initial introduction to prefectures. This trend may reflect that first year had a lot of training and capacity building and support while second year the lab staff need to understand and execute the operating procedures independently until they had good command. The average time for solid culture and DST continuously decreased after 2016, but it was still two times longer than the rapid molecular DST. Therefore, the application of the rapid molecular DST can reduce diagnosis delay and enable prompt treatment.

We observed significant changes across various indicators after the implementation of DR-TB detection interventions. The proportion of bacteriologically confirmed cases tested for drug resistance varied greatly across prefectures in 2013. Some prefectures, particularly those with the poorest progress in DR-TB detection, experienced rapid increases between 2016 and 2019 (Yangzhou and Huai’an). Not as international projects that only made progress in project sites, the implementation of provincial policies on DR-TB improved the DR-TB detection across all prefectures after 2017. However, most indicators assessed did not have a linear trend of improvement, indicating the complexity of policy interventions affected by other factors such as domestic migration, economic development, technical capacity of health facilities, or different health insurance policies applied in different regions of Jiangsu.

In additional to findings about the improved detection of TB by application of RMT, our regression models also found that males, adults (under 60), new TB patients, those without a cavity in a lung, and those residing within a county were less likely to be bacteriologically confirmed for TB, compared to their counterparts. Prefecture-level or specialized hospitals, CDC, or TB dispensaries were less likely to provide bacteriological diagnosis for TB. For drug resistance testing, our findings indicate that patients not biologically confirmed for TB, smear negative or culture positive, male, adults (under 60), new patients, and those residing outside the county, were less likely to be tested for drug resistance. Prefecture-level, specialized hospitals, CDC, or TB dispensary were less likely to provide drug resistance tests. These findings indicate the potential policy gaps that these sub-groups of the population may need more attention to receive proper TB diagnosis.

This is an observational study that uses routine administrative data to understand the effects of DR-TB policy interventions across different regions of Jiangsu Province between 2013 and 2019. We attempted to capture the impact of a group of interventions that have been implemented in various combinations and sequences. By using the TBIMS to assess the screening and detection of TB, we explored the potential of using this routine data to regularly monitor TB related outcomes and inform TB policy in China.

Our study faced some limitations. First, random control design was not possible since this study relied on routine, retrospective data. Second, we were only able to analyse indicators already collected by the TBIMS. Other factors impacting the results (e.g., the insurance status of TB patients) were not fully recognized or reflected in the analysis. Third, the TBIMS was originally designed for case reporting and disease monitoring. After the implementation of different policies, the overall quality of data from TBIMS has been improved and the DR-TB data module was developed in recent years. However, data from TBIMS hasn’t been fully used for monitoring quality of TB services. It does not necessarily have robust quality assurance, and errors in data entry cannot be ruled out. Improvements to the quality of TBIMS will benefit the further policy development for TB control and care in China.

## Conclusions

To fully scale up DR-TB detection across the country, long-term sustainable policies at the provincial, or even the national level, are needed. The application of new diagnostic devices and expanded screening strategy have improved the detection of DR-TB patients. Along with these improvements in DR-TB detection, sufficient resources and strong political commitment from the local government have played an important role in improving the implementation of DR-TB detection.

## Supplementary Information

**Additional file 1.** Supplementary files. **1**. Socioeconomics in study sites. **2**. Workforce and funding for TB. **3**. Process charts for TB testing in Jiangsu.

## Data Availability

The datasets analysed during the current study are not publicly available due the regulation on TBIMS but are available from the corresponding author on reasonable request.

## Abbreviations

BMU: Basic management unit
CDC: Center for disease control and prevention
DR-TB: Drug-resistant tuberculosis
DST: Drug susceptibility testing
Global Fund: The Global Fund to Fight AIDS, Tuberculosis and Malaria
HIS: Hospital information system
IA: Isothermal amplification technology
MDR-TB: Multidrug-resistant tuberculosis
RMT: Rapid molecular tests
RR-TB: Rifampicin-resistant tuberculosis
TB: Tuberculosis
TBIMS: TB information management system
