# Supplementary material for: Impact of multiple policy interventions on the screening and diagnosis of drug-resistant tuberculosis patients: a cascade analysis on six prefectures in China
Source: Infect Dis Poverty. 2021 Jan 19;10:8. doi: 10.1186/s40249-021-00793-9 (PMC7814633; doi:10.1186/s40249-021-00793-9)
Supplement: Supplementary file 1 — Additional file 1. Supplementary files. 1. Socioeconomics in study sites. 2. Workforce and funding for TB. 3. Process charts for TB testing in Jiangsu. [file 40249_2021_793_MOESM1_ESM.docx]

**Supplementary files for Impact of multiple policy interventions on the screening and diagnosis of drug-resistant tuberculosis patients: a cascade analysis on six prefectures in China**

## **Supplementary file 1 Socioeconomics in study sites**

| **Prefecture** | **Number of counties** | | **Permanent resident population* (1000)** | | **Proportion of urban population （%）** | | **GDP （billion, USD）** | | **Average disposable income (USD) - Urban** | | **Average disposable income (USD) – Rural** | |
| --- | --- | --- | --- | --- | --- | --- | --- | --- | --- | --- | --- | --- |
|  | **2013** | **2018** | **2013** | **2018** | **2013** | **2018** | **2013** | **2018** | **2013** | **2018** | **2013** | **2018** |
| **Zhenjiang** | 6 | 6 | 3165 | 3196 | 65.4 | 71.2 | 47.3 | 61.4 | 5319 | 7410 | 2622 | 3740 |
| **Changzhou** | 7 | 6 | 4692 | 4729 | 67.5 | 72.5 | 70.3 | 106.8 | 5905 | 8182 | 3007 | 4245 |
| **Huai’an** | 8 | 7 | 4827 | 4925 | 55.1 | 62.4 | 34.8 | 54.5 | 3727 | 5428 | 1781 | 2585 |
| **Lianyungang** | 7 | 6 | 4428 | 4520 | 55.7 | 62.6 | 28.9 | 42.0 | 3707 | 4962 | 1735 | 2516 |
| **Nantong** | 8 | 8 | 7298 | 7310 | 59.9 | 67.1 | 81.3 | 127.7 | 5082 | 7018 | 2380 | 3389 |
| **Yangzhou** | 6 | 6 | 4470 | 4531 | 60.0 | 67.1 | 52.4 | 82.9 | 4540 | 6363 | 2293 | 3251 |

*Permanent resident population were estimated through resident population at year-end

** In 2013, 1 USD = 6.2 CNY; In 2018, 1 USD = 6.6 CNY.

## **Supplementary file 2Workforce and funding for TB**

| **Prefecture** | **Number of designated TB hospital** | | **Number of RMT devices** | | **Number of full-time staff of CDC** | | **Number of full-time staff of designated TB hospital** | | **Special funds for MDR/RR-TB**  **(1000 USD)** | |
| --- | --- | --- | --- | --- | --- | --- | --- | --- | --- | --- |
|  | **2013** | **2018** | **2013** | **2018** | **2013** | **2018** | **2013** | **2018** | **2013** | **2018** |
| Zhenjiang | 5 | 4↓ | 1 | 5↑ | 26 | 21↓ | 71 | 61↓ | 4.8 | 29.8 ↑ |
| Changzhou | 4 | 4→ | 0 | 4↑ | 16 | 15↓ | 10 | 10→ | 34.8 | 40.9 ↑ |
| Huai’an | 7 | 7→ | 0 | 7↑ | 17 | 17→ | 15 | 21↑ | 16.9 | 45.0 ↑ |
| Lianyungang | 5 | 5→ | 1 | 5↑ | 20 | 18↓ | 19 | 21↑ | 51.5 | 42.9 ↓ |
| Nantong | 7 | 7→ | 1 | 8↑ | 26 | 21↓ | 76 | 82↑ | 4.8 | 59.7 ↑ |
| Yangzhou | 5 | 5→ | 0 | 5↑ | 15 | 14↓ | 32 | 71↑ | 23.2 | 43.2 ↑ |
| **Provincial level** |  |  |  |  |  |  |  |  | 208.1 | 878.8 ↑ |

*In 2013, 1 USD = 6.2 CNY; In 2018, 1 USD = 6.6 CNY. RMT: Rapid molecular testing; CDC: Center for Disease Prevention and Control; MDR-TB: multidrug-resistant TB; RR-TB: Rifampicin-resistant tuberculosis

## **Supplementary file 3 Process Charts for TB testing in Jiangsu**

Sputum smear microscopy

Negative

Positive

Culture

Negative

Positive

Drug susceptibility testing (proportion method)

RR-TB

MDR-TB

XDR-TB

**County level**

**Prefecture level**

**Appendix 3a Process chart with proportion method before 2017**

RR-TB: Rifampicin-resistant tuberculosis; MDR-TB: multidrug-resistant TB; XDR-TB: extensively drug-resistant tuberculosis.

Sputum smear microscopy

Negative

Positive

Culture

Negative

Positive

Drug susceptibility testing (GeneChip or Hain)

Second line drug susceptibility test

MDR/RR-TB

**County level**

**Prefecture level**

**Appendix 3b Process chart with rapid molecular testing before 2017**

RR-TB: Rifampicin-resistant tuberculosis; MDR-TB: multidrug-resistant TB.

Presumptive TB

Sputum smear microscopy + chest X ray

Non-TB patients

Smear-positive TB

Smear-negative TB

Rapid molecular testing

(Xpert)

Culture

Positive

Negative

Drug susceptibility test

MDR/RR-TB

Second line drug susceptibility test

Rifampin sensitive TB and others

RR-TB

**County level**

**Prefecture level**

**Appendix 3c Process chart of Zhenjiang and Nantong prefectures after 2017**

RR-TB: Rifampicin-resistant tuberculosis; MDR-TB: multidrug-resistant TB.

Presumptive TB

Sputum smear microscopy+ chest X ray

Non-TB patients

Smear-positive TB

Smear-negative TB

Rapid molecular testing (LAMP)

Culture

Positive

Negative

Drug susceptibility test

MDR/RR-TB

Second line drug susceptibility test

Positive

Negative

**County level**

**Prefecture level**

**Appendix 3d Process chart of Changzhou, Yangzhou, Lianyungang and Huai’an prefectures after 2017**

RR-TB: Rifampicin-resistant tuberculosis; MDR-TB: multidrug-resistant TB.
